# Benign Prostatic Hyperplasia and the Risk of Prostate Cancer and Bladder Cancer: A Meta-Analysis of Observational Studies

**DOI:** 10.1097/MD.0000000000003493

**Published:** 2016-05-06

**Authors:** Xiaoyu Dai, Xiangming Fang, Ying Ma, Jianbo Xianyu

**Affiliations:** From the Department of Nephrology (XD); Department of Endocrinology (XF); Department of Gynaecology and Obstetrics (MY); Department of General Surgery (JX), Mianyang Central Hospital, Mianyang, China.

## Abstract

Benign prostatic hyperplasia (BPH) has been suggested to be a risk factor for certain urologic cancers, but the current evidence is inconsistent.

The aim of this study was to investigate the association between BPH and urologic cancers.

MEDLINE, EMBASE, Cochrane Library, and Web of Science were searched for potential eligible studies.

We included case-control studies or cohort studies, which evaluated the association between BPH and urologic cancers (including prostate cancer, bladder cancer, kidney cancer, testicular cancer, or penile cancer).

Overall effect estimates were calculated using the DerSimonian–Laird method for a random-effects model. Summary effect-size was calculated as risk ratio (RR), together with the 95% confidence interval (CI).

This systematic review included 16 case-control studies and 10 cohort studies evaluating the association of BPH and prostate or bladder cancer; we did not identify any study about other urologic cancers. Meta-analyses demonstrated that BPH was associated with an increased incidence of prostate cancer (case-control study: RR = 3.93, 95% CI = 2.18–7.08; cohort-study: RR = 1.41, 95% CI = 1.00–1.99) and bladder cancer (case-control study: RR = 2.50, 95% CI = 1.63–3.84; cohort-study: RR = 1.58, 95% CI = 1.28–1.95). Subgroup analysis by ethnicity suggested that the association between BPH and prostate cancer was much stronger in Asians (RR = 6.09, 95% CI = 2.96–12.54) than in Caucasians (RR = 1.54, 95% CI = 1.19–2.01). Egger's tests indicated low risk of publication bias (prostate cancer: *P* = 0.11; bladder cancer: *P* = 0.95).

BPH is associated with an increased risk of prostate cancer and bladder cancer. The risk of prostate cancer is particularly high in Asian BPH patients. Given the limitations of included studies, additional prospective studies with strict design are needed to confirm our findings.

## Abstract 2

Supplemental Digital Content is available in the text

## INTRODUCTION

Benign prostatic hyperplasia (BPH) is a nonmalignant enlargement of the prostate caused by cellular hyperplasia.^1^ It is a common age-associated disease affecting ∼70% of men aged 70 years or over.^2^ BPH can be a bothersome and potentially severe condition. Not only can it lead to lower urinary tract symptoms (LUTS) and diminish patients’ quality of life,^3^ but it may also be associated with certain male urologic cancers such as prostate cancer^4^ and bladder cancer.^5,6^ The mechanism between BPH and urologic cancers is not fully understood. Some studies suggested that hormones, inflammation, metabolic syndrome are likely to play a role in BPH and prostate cancer.^7^ For bladder cancer, a possible explanation for the association is that the residual urine in the bladder in patients with BPH may cause lower urinary tract damage and BPH may prolong the time of urothelial exposure to urinary excreted carcinogens.^8^

To date many epidemiological studies have investigated the association between BPH and prostate cancers, which is one of the most common cancers worldwide and the number one cause of cancer death for men in the developed countries.^9^ However, the findings of these studies are inconsistent.^10–12^ Similarly, past studies investigating bladder cancer risk in BPH patients also gave inconsistent results.^5,6,13,14^ The evidence of the association between BPH and the risk of urologic cancers other than prostate and bladder cancers is lacking and seldom reviewed systematically. Owing to the high prevalence of BPH and urologic cancers, investigation of their association is of great public health and clinical significance. Knowledge of this link could enable physicians to take common preventative strategies for BPH and urologic cancers, to improve the effectiveness of cancer screening, and potentially to treat cancer at an earlier stage.^7,15^ To the best of our knowledge, there is no systematic review that studied the association between BPH and urologic cancer risk. We, therefore, performed this meta-analysis to estimate the magnitude of any association between BPH and male urologic cancers. Our null hypothesis was that the urologic cancer risk was equal between people with or without BPH.

## METHODS

This study was performed and reported according to the recommendation of Meta-analysis Of Observational Studies in Epidemiology (MOOSE) group.^16^ Ethics approval was not need as this is a secondary literature-based study.

### Inclusion and Exclusion Criteria

Studies were eligible for inclusion if they meet the following criteria: (1) the study was undertaken in general population or in patients with certain disease such as diabetes; (2) the exposure of interest was BPH, which can be defined by different criteria such as International Classification of Diseases; (3) the primary outcomes were urologic cancers including prostate cancer, bladder cancer, kidney cancer, testicular cancer, and/or penile cancer; (4) the study design was case-control studies and cohort studies. We excluded studies which did not report the adjusted effect or raw data about the association between BPH and urologic cancers. Cross-sectional studies, randomized clinical trials, and other study design were not eligible for inclusion. If multiple published reports were from the same study cohort, we included only the 1 with the most detailed information for both coffee consumption and outcome.

### Literature Searches

MEDLINE (inception ∼ May 2015), EMBASE (inception ∼ May 2015), Cochrane Library (inception ∼ March 2016), and Web of Science (inception ∼ March 2016) were searched to identify eligible studies. The search strategy consisted of search items for BPH, urologic cancers, and observational studies with the following subject headings and the text keywords: “benign prostatic hyperplasia”, “prostate cancer”, “bladder cancer”, “kidney cancer”, “testicular cancer”, “penile cancer”, “case-control study”, and “cohort study”. All the searches were restricted to human studies and there was no limitation on publication status or language. Bibliographies of the included studies and relevant review articles were manually checked to identify additional studies.

### Study Selection

All the citations obtained from literature searches were initially downloaded into reference management software and the duplicated citations were electronically removed. Two authors then independently evaluated the eligibility of remaining studies by examining the titles, abstracts, and full articles sequentially, with discrepancies resolved by discussion.

### Data Extraction and Quality Assessment

Two authors independently extracted the following information from included studies: study information, patient characteristics, matched or adjusted factors, information for study quality assessment, and estimated effects. We consulted the authors of original studies to collect missing information as necessary. Two authors independently evaluated the quality of included studies with the Newcastle–Ottawa Scale (NOS), which is a 9-score system assessing the risk of bias from participant selection, comparability, and exposure or outcome.^17^

### Statistical Analysis

Regarding the relative risk (RR), different measures of estimated effect were recorded or calculated, including odds ratio for case-control studies, risks ratio, or hazard ratio for cohort studies. We assumed that all of these effect measures would give a similar effect estimate and they were considered equally in the overall effect estimate.^18^

For each study, the RR and its corresponding standard error were transformed to the natural logarithms to stabilize the variance and to normalize the distributions. We calculated the variance in each study's measure of effect from the 95% confidence intervals (CIs). When both crude and adjusted RRs were provided, we used the most fully adjusted value. Overall effect estimates were calculated using the DerSimonian–Laird method for a random-effects model that considered both within- and between-study variation.^19^ Heterogeneity among studies was assessed with the *Q*-test and the *I*^2^ -index statistic. The low level of heterogeneity was defined as *I*^2^ ≤ 25%, accompanied by *P* > 0.10 for the *Q*-test.^20^ Where significant heterogeneity was identified, we investigated the source of heterogeneity by subgroup analyses and meta-regression. The potential source of heterogeneity we investigated includes study design (case-control study and case-control study), ethnicity (Caucasian and Asian), source of participants (population-based study and hospital-based study), and quality of included studies (NOS score >6 and NOS score ≤6).

Sensitivity analyses were carried out by excluding single-arm cohort studies, studies with high risk of bias, studies performed in patients with diabetes mellitus, or studies with a confirmed definition of BPH (confirmed by International Classification of Diseases or by a prior history of surgery or medical treatment for BPH). Publication bias was examined by funnel plots and Egger's test. Data analyses were undertaken using Review Manager (RevMan 5.2.9) and STATA 12 (STATA, College Station, TX). The study was reported according to the recommendation of Meta-analysis of Observational Studies in Epidemiology Group.^16^

## RESULTS

### Study Characteristics

**FIGURE 1:**
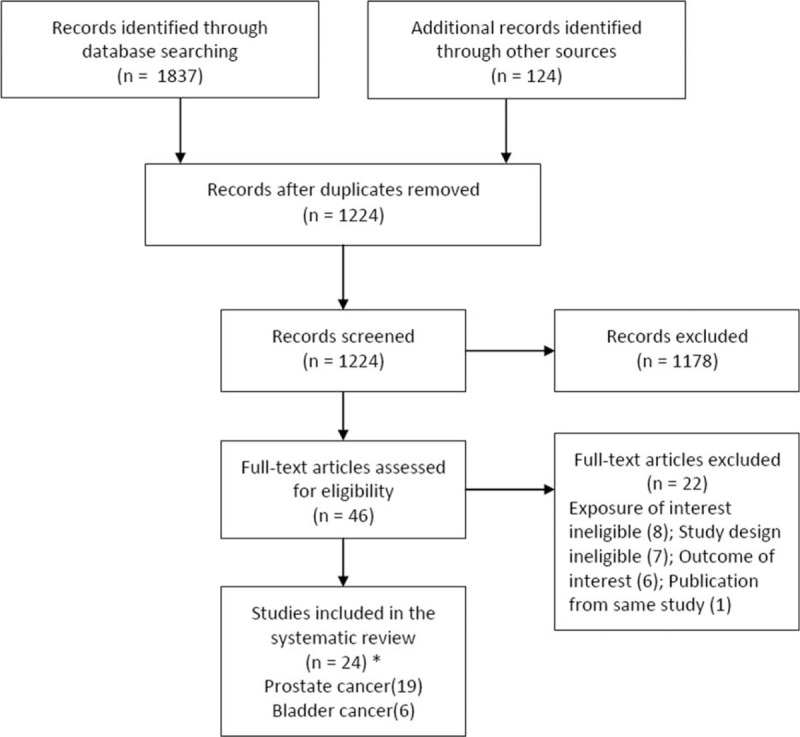
Flowchart of study selection. ^∗^One cohort study investigated both prostate cancer and bladder cancer, and 1 study reported both prospective and retrospective data.

The literature search yielded 1961 potentially relevant citations, of which 737 duplicates were excluded and 1224 citations were removed after reviewing titles and abstracts. The full texts of 46 remaining citations were screened, and finally 24 studies ^S1–S24^ including 1,615,099 participants were included (Figure 1, Supplemental digital content—Text). Nineteen^S1–S3,^^S6–S10,^^S12,^^S14–S20,^^S22,^^S23^ and 6^S4–S6,^^S11,^^S13,^^S24^ studies investigated the risk of prostate cancer and bladder cancer, respectively, with 1 cohort study ^S6^ considering both outcomes of interest. We did not identify any study evaluating the risk of BPH and the risk of urologic cancer other than prostate cancer and bladder cancer.

Regarding prostate cancer, 14 studies used a case-control design^S7–S10,^^S12,^^S15–S24^, 6 were cohort studies,^S1–S3,^^S6,^^S14,^^S16^ and 1 publication reported both prospective and retrospective data^S16^. For the 6 studies that investigated risk of bladder cancer, 2 were case-control studies^S11,^^S24^ and 4 were cohort studies^S4–S6,^^S13^. Three studies were carried out in Caucasians,^S4,^^S6,^^S11^ 2 in Asians,^S5,^^S24^ and 1 did not report the race of the participants.^S13^ The detailed characteristics of included studies were presented in Tables 1 and 2.

**TABLE 1:**
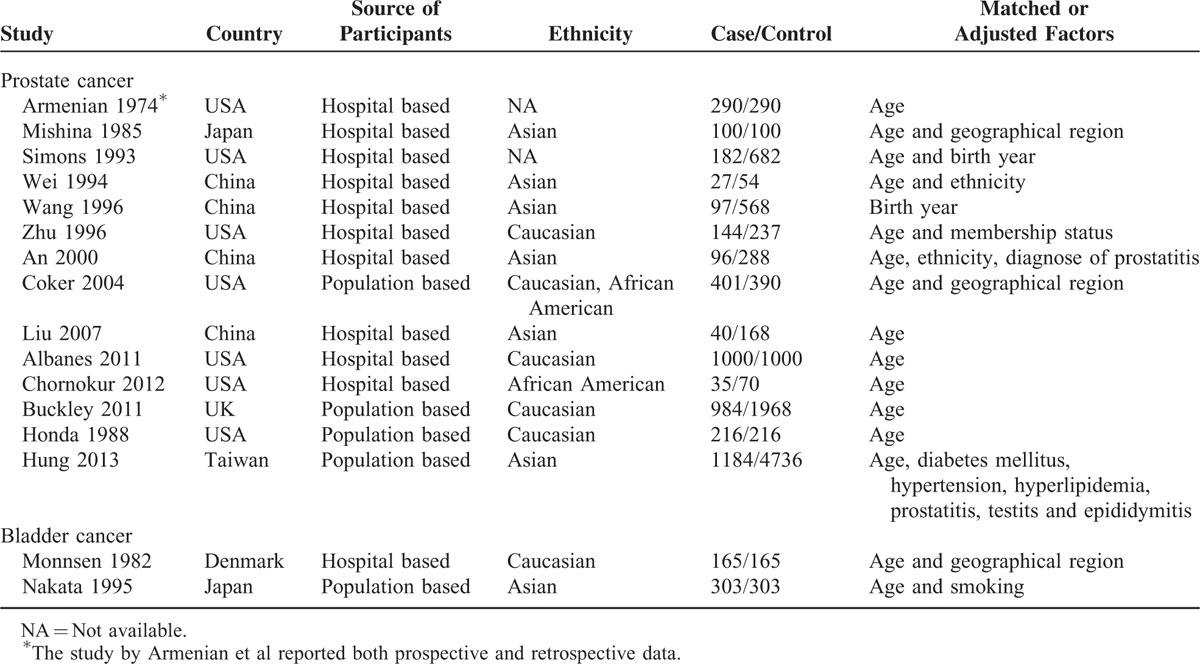
Characteristics of Included Case-Control Studies

**TABLE 2:**
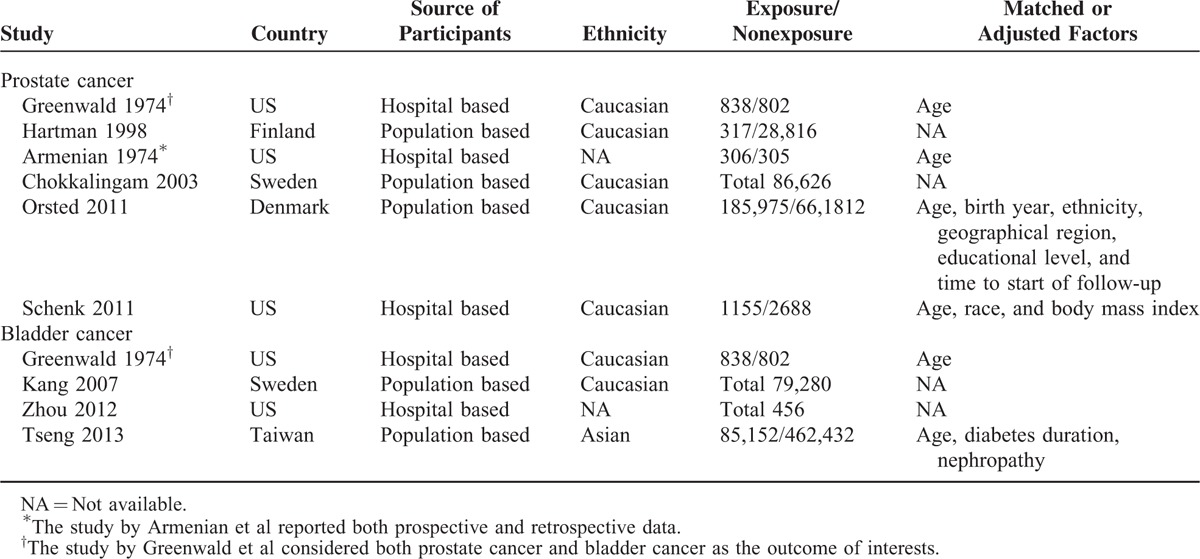
Characteristics of Included Cohort Studies

### Quality of Included Studies

Overall, the quality of included studies was moderate, with 20 studies achieving an NOS score over 6 points. In terms of the case-control studies, all were adequate in case definition, no participants in the control group had a history of prostate cancer (or bladder cancer). Nine studies^S9,^^S10,^^S12,^^S16,^^S17,^^S19,^^S21,^^S23,^^S24^ included consecutive or obviously representative series of cases and 6 studies ^S8,^^S10,^^S20,^^S21,^^S23,^^S24^ selected community controls. All of the included studies controlled or adjusted for age or other important factors on the basis of study design or analysis. Two studies^S18,^^S19^ had no description of the ascertainment of exposure and 7 studies^S9,^^S16,^^S18,^^S19,^^S21,^^S22,^^S24^ had the same nonresponse rates for both study groups.

As for the 9 included cohort studies, 7^S1–S3,^^S5,^^S6,^^S13,^^S14^ were considered to be truly or somewhat representative of BPH patients in the community and 6 studies^S2–S6,^^S16^ selected the nonexposed cohort from the same community as the exposed cohort. The ascertainment of exposure was not described in 1 study^S13^ and 7 studies^S2–S4,^^S6,^^S13,^^S14,^^S16^ demonstrated that participants were cancer free at the start of the study. All of the included studies controlled or adjusted for age or other important factors on the basis of the design or analysis. The follow-up was adequate and long enough for outcomes to occur in all the studies. The detailed evaluation of risk of bias was reported in the Supplemental digital content—Tables 1 and 2.

### BPH and Risk of Prostate Cancer

**FIGURE 2:**
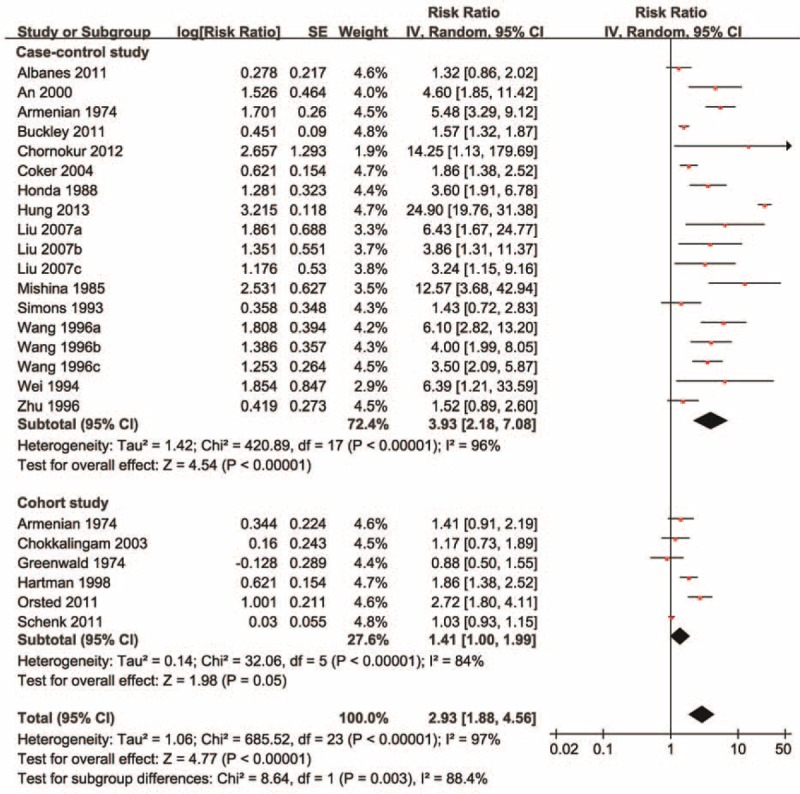
The relative risk of prostate cancer in men with benign prostatic hyperplasia. The diamonds indicated the pooled relative risks; the size of each box indicates the relative weight of each study in the meta-analysis; the horizontal bars show the 95% confidence intervals. CI = confidence interval.

Meta-analysis of all 19 studies demonstrated that BPH was associated with an increased risk of prostate cancer (RR = 2.93, 95% CI = 1.88–4.56), with significant between-study heterogeneity (*I*^2^ = 97%; *P* < 0.00001) (Figure 2 and Table 3). Subgroup analysis by study design showed that the association between BPH and prostate cancer was stronger in case-control studies (RR = 3.93, 95% CI = 2.18–7.08) than in cohort studies (RR = 1.41, 95% CI = 1.00–1.99), with a significant subgroup difference (*P* = 0.003). Subgroup analysis by ethnicity suggested that the association between BPH and prostate cancer was stronger in Asians (RR = 6.09, 95% CI = 2.96–12.54) than in Caucasians (RR = 1.54, 95% CI = 1.19–2.01). The difference remained significant in the meta-analysis of case-control studies (Asians: RR = 6.09, 95% CI = 2.96–12.54; Caucasians: RR = 1.79, 95% CI = 1.26–2.55). Subgroup analysis by ethnicity was not undertaken among cohort studies as there was no cohort study carried out in Asians. Subgroup analysis by the source of participant recruitment (*P* = 0.71) and study quality (*P* = 0.78) indicated no significant subgroup differences (Table 3).

**TABLE 3:**
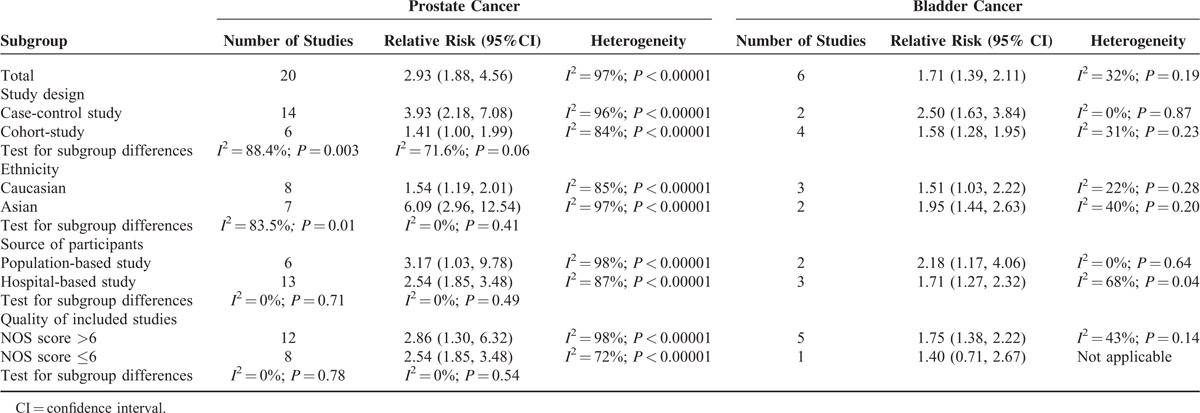
Meta-Analysis of Benign Prostatic Hyperplasia and the Risk of Urologic Cancer

The subgroup analysis results were confirmed by univariate meta-regressions according to ethnicity (*P* < 0.001; adjusted *R*-squared = 60.43%), study design (*P* = 0.011; adjusted *R*-squared = 23.69%), and study quality (*P* = 0.011; adjusted *R*-squared = 0%). Multivariate meta-regression suggested that it was ethnicity (*P* = 0.004; adjusted *R*-squared = 58.11%) rather than study design (*P* = 0.555; adjusted *R*-squared = 58.11%) that was significantly associated with the estimated effects.

### BPH and Risk of Bladder Cancer

**FIGURE 3:**
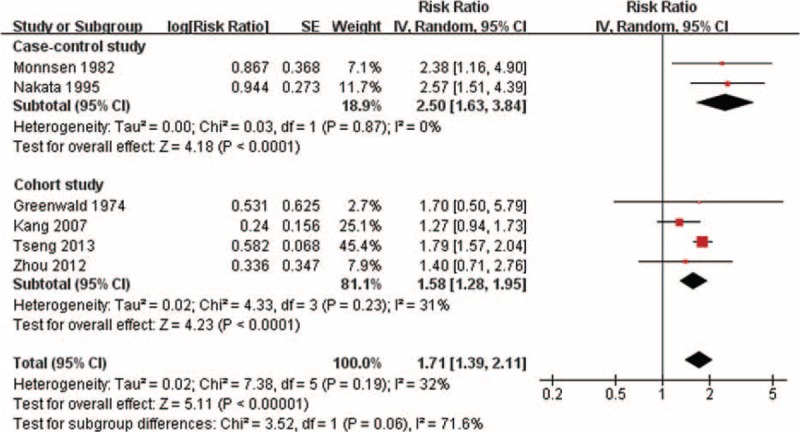
The relative risk of bladder cancer in men with benign prostatic hyperplasia. The diamonds indicated the pooled relative risks; the size of each box indicates the relative weight of each study in the meta-analysis; the horizontal bars show the 95% confidence intervals. CI = confidence interval.

Mata-analysis including all 6 studies indicated that BPH was associated with an increased risk of bladder cancer (RR = 1.71, 95% CI = 1.39–2.11), with no significant between-study heterogeneity (*I*^2^ = 32%; *P* = 0.19) (Table 3 and Figure 3). The association of BPH and bladder cancer tended to be stronger in case-control studies (RR = 2.50, 95% CI = 1.63–3.84) than that in cohort studies (RR = 1.58, 95% CI = 1.28–1.95). Subgroup analyses by ethnicity, source of participant recruitment, and study quality generally showed a significantly increased risk of cancer with BPH in the subgroups, with no significant subgroup differences (Table 3). Meta-regression was not performed as only 6 studies were included.

### Publication Bias and Sensitivity Analysis

A visual inspection showed some asymmetry in the funnel plot for prostate cancer but none in the funnel plot for bladder cancer (Supplemental digital content—Figure). Egger's tests indicated no significant publication bias (prostate cancer: *P* = 0.11; bladder cancer: *P* = 0.95).

For both prostate cancer and bladder cancer, sensitivity analyses by excluding studies with high risk of bias, studies without matching or adjusting for any potentially confounding factor, or single-arm cohort studies did not show major influence to the estimated effects. BPH remained a significant risk factor for bladder cancer after excluding the studies performed diabetes patients (Supplemental digital content—Table 3).

## DISCUSSION

In this systematic review of observational studies, we found a positive, significant association between BPH and the incidence of prostate cancer/bladder cancer, both in the meta-analysis including all eligible studies and in the subgroup analyses for various factors. Overall, BPH was associated with ∼2.9-fold increased incidence of prostate cancer and 1.7-fold increased incidence of bladder cancer. Meta-analyses of the association between BPH and risk of prostate cancer show significant between-study heterogeneity. This may partly due to the difference in ethnicity. The associated risk of prostate cancer seems to be much larger in Asians than in Caucasians.

In addition to epidemiological evidence, accumulating evidence indicates that hormones,^7^ inflammation,^21,22^ metabolic syndrome ^23,24^ are likely to play a role in BPH and prostate cancer. The homeostasis between prostate cell proliferation and cell death supported by dihydrotestosterone and estrogen is often disrupted in BPH patients.^25^ Fast-growing BPH is associated with an increased risk of prostate cancer and an increased likelihood that such cancer will be high grade.^24^ Inflammation is a well-established risk factor for both BPH and prostate cancer. Some studies have indicated that inflammatory infiltrates were more likely to suffer progression from BPH to prostate cancer than those without inflammation.^20,29^ There is evidence that both BPH and prostate cancer are components of the metabolic syndrome.^23^ Many studies have also linked the metabolic syndrome with fast-growing BPH, which is a strong risk factor for the development of prostate cancer.^24^

In terms of the association between BPH and bladder cancer, current evidence supports the notion of a causal link. A possible explanation for the association is that the residual urine in the bladder in patients with BPH may cause lower urinary tract damage, and moreover, BPH may also prolong the time of urothelial exposure to urinary excreted carcinogens.^8,26,27^ This was indirectly supported by prospective evidence that high fluid intake, indicating less concentrated urine, was associated with lower risk of bladder cancer.^27^

Given the differences in prevalence, prognosis, and survival between Asian and Caucasian prostate cancer patients,^2,28^ it is reasonable to suspect that the mechanism through which BPH contribute to prostate cancer maybe different between populations. In some case-control studies of Asian males, the relative risks can be as high as 13 to 26.^S12,^^S21^ The large estimates of the magnitude of effect can improve the quality of evidence.^29^ This is of great importance for the management of Asian BPH patients.

An important threat to the validity of our study finding is BPH detection bias in patients, particularly for the prostate cancer risk. It has been shown that increased disease awareness in BPH patients may increase the likelihood of being diagnosed with prostate cancer.^26^ In the study by Schenk et al,^12^ the authors found no significant association between BPH and prostate cancer incidence. The risk of detection bias in this study is low as all the participants received the same annual clinical examinations. However, this study was performed in a highly selected population and the generalizability of the findings is low. To the contrary, Orsted and colleagues found a positive association between BPH and prostate cancer incidence as well as mortality in the largest population-based cohort study.^4^ The association was still significant in the sensitivity analyses by excluding participants who had a diagnosis of prostate cancer within 1, 5, or 10 years after the start of follow-up, or by the introduction of prostate-specific antigen test. These statistical analyses minimized the potential influence from detection bias and suggested that detection bias alone cannot explain the positive link between BPH and prostate cancer.

Other limitations of this systematic review primarily arise from the risk of bias in the original studies. Some of the included studies may have patient selection bias or bias in ascertainment of exposures and outcomes. However, subgroup analysis and sensitivity analysis according to study quality did not reveal potential explanations for estimate effects. Additionally, the heterogeneity in the meta-analysis of prostate cancer may reduce the precision of the estimates. We applied a random-effects model and performed subgroup analysis to minimize the potential influence. Moreover, the definition of BPH was not clearly reported in many studies, so it is unclear whether clinical BPH increased the likelihood of cancer diagnosis.

To the best of our knowledge, this study is the first and most comprehensive study of BPH and the risk of urologic cancer. An exhaustive search of up-to-date literature was undertaken to ensure that most eligible observational studies were included. In addition, the total number of participants contributed to data analysis is much larger than any of the past studies on this topic. This allowed us to carry out stratified analysis to investigate the potential influential factor. Lastly, the robust sensitivity analyses and consistency in the subgroup analyses lend strength to our confidence in the study conclusion.

In conclusion, BPH is associated with an increased incidence for both prostate cancer and bladder cancer, and the prostate cancer risk is particularly high in Asian BPH patients. These findings can provide evidence in guiding cancer prevention and screening. Given the limitations of included studies, particularly for detection bias, additional prospective studies with strict design are needed to confirm our findings.

## Supplementary Material

Supplemental Digital Content

## References

[1] Chapple C Medical treatment for benign prostatic hyperplasia. *BMJ* 1992; 304:1198–1199. 1381250 10.1136/bmj.304.6836.1198 PMC1881763

[2] McVary K BPH: epidemiology and comorbidities. *Am J Manag Care* 2006; 12:S122–128. 16613526

[3] McVary KT Roehrborn CG Avins AL Update on AUA guideline on the management of benign prostatic hyperplasia. *J Urol* 2011; 185:1793–1803. 21420124 10.1016/j.juro.2011.01.074

[4] Orsted D Bojesen S Nielsen S Association of clinical benign prostate hyperplasia with prostate cancer incidence and mortality revisited: a nationwide cohort study of 3 009 258 men. *Eur Urol* 2011; 60:691–698. 21705134 10.1016/j.eururo.2011.06.016

[5] Tseng C Benign prostatic hyperplasia is a significant risk factor for bladder cancer in diabetic patients: A population-based cohort study using the National Health Insurance in Taiwan. *BMC Cancer* 2013; 13:7. 23286275 10.1186/1471-2407-13-7 PMC3541059

[6] Mommsen S Aagaard J Sell A An epidemiological case-control study of bladder cancer in males from a predominantly rural district. *Eur J Cancer Clin Oncol* 1982; 18:1205–1210. 6897634 10.1016/0277-5379(82)90103-1

[7] Alcaraz A Hammerer P Tubaro A Is There evidence of a relationship between benign prostatic hyperplasia and prostate cancer? Findings of a literature review. *Eur Urol* 2009; 55:864–875. 19027219 10.1016/j.eururo.2008.11.011

[8] Kadlubar F Dooley K Teitel C Frequency of urination and its effects on metabolism, pharmacokinetics, blood hemoglobin adduct formation, and liver and urinary bladder DNA adduct levels in beagle dogs given the carcinogen 4-aminobiphenyl. *Cancer Res* 1991; 51:4371–4377. 1868460

[9] Siegel R Naishadham D Jemal A Cancer statistics, 2012. *CA Cancer J Clin* 2012; 62:10–29. 22237781 10.3322/caac.20138

[10] HK A AM L EL D Relation between benign prostatic hyperplasia and cancer of the prostate. A prospective and retrospective study. *Lancet* 1974; 2:115–117. 4135500 10.1016/s0140-6736(74)91551-7

[11] Chokkalingam A Nyren O Johansson J Prostate carcinoma risk subsequent to diagnosis of benign prostatic hyperplasia: a population-based cohort study in Sweden. *Cancer* 2003; 98:1727–1734. 14534890 10.1002/cncr.11710

[12] Schenk JM Kristal AR Arnold KB Association of symptomatic benign prostatic hyperplasia and prostate cancer: results from the prostate cancer prevention trial. *Am J Epidemiol* 2011; 173:1419–1428. 21540324 10.1093/aje/kwq493 PMC3276227

[13] Greenwald P Kirmss V Polan A Cancer of the prostate among men with benign prostatic hyperplasia. *J Natl Cancer Inst* 1974; 53:335–340. 4843266 10.1093/jnci/53.2.335

[14] Kang D Chokkalingam A Gridley G Benign prostatic hyperplasia and subsequent risk of bladder cancer. *Brit J Cancer* 2007; 96:1475–1479. 17473820 10.1038/sj.bjc.6603730 PMC2360186

[15] Orsted DD Bojesen SE The link between benign prostatic hyperplasia and prostate cancer. *Nat Rev Urol* 2013; 10:49–54. 23165396 10.1038/nrurol.2012.192

[16] Stroup DF Berlin JA Morton SC Meta-analysis of observational studies in epidemiology: a proposal for reporting. Meta-analysis of Observational Studies in Epidemiology (MOOSE) group. *JAMA* 2000; 283:2008–2012. 10789670 10.1001/jama.283.15.2008

[17] Rostom A Wells G Tugwell P Prevention of NSAID-induced gastroduodenal ulcers. *Cochrane Database Syst Rev* 2000; CD002296. 10.1002/14651858.CD002296 11034748

[18] Greenland S Quantitative methods in the review of epidemiologic literature. *Epidemiol Rev* 1987; 9:1–30. 3678409 10.1093/oxfordjournals.epirev.a036298

[19] DerSimonian R Laird N Meta-analysis in clinical trials. *Control Clin Trials* 1986; 7:177–188. 3802833 10.1016/0197-2456(86)90046-2

[20] Higgins J Thompson S Quantifying heterogeneity in a meta-analysis. *Stat Med* 2002; 21:1539–1558. 12111919 10.1002/sim.1186

[21] Roehrborn C Kaplan S Noble W The impact of acute or chronic inflammation in baseline biopsy on the risk of clinical progression of BPH: results from the MTOPS study. *J Urol* 2005; 173:346.

[22] MacLennan GT Eisenberg R Fleshman RL The influence of chronic inflammation in prostatic carcinogenesis: a 5-year followup study. *J Urol* 2006; 176:1012–1016. 16890681 10.1016/j.juro.2006.04.033

[23] Ozden C Ozdal OL Uryancioglu G The correlation between metabolic syndrome and prostatic growth in patients with benign prostatic hyperplasia. *Eur Urol* 2007; 51:199–206. 16806666 10.1016/j.eururo.2006.05.040

[24] Hammarsten J Hogstedt B Clinical, haemodynamic, anthropometric, metabolic and insulin profile of men with high-stage and high-grade clinical prostate cancer. *Blood Press* 2004; 13:47–55. 15083641 10.1080/08037050310025735

[25] Hendriksen PJM Dits NFJ Kokame K Evolution of the androgen receptor pathway during progression of prostate cancer. *Cancer Res* 2006; 66:5012–5020. 16707422 10.1158/0008-5472.CAN-05-3082

[26] Jacobsen SJ Girman CJ Guess HA Do prostate size and urinary flow rates predict health care-seeking behavior for urinary symptoms in men? *Urology* 1995; 45:64–69. 7529448 10.1016/s0090-4295(95)96766-4

[27] Michaud DS Spiegelman D Clinton SK Fluid intake and the risk of bladder cancer in men. *N Engl J Med* 1999; 340:1390–1397. 10228189 10.1056/NEJM199905063401803

[28] Kimura T East meets West: ethnic differences in prostate cancer epidemiology between East Asians and Caucasians. *Chin J Cancer* 2012; 31:421–429. 22085526 10.5732/cjc.011.10324 PMC3777503

[29] Guyatt GH Oxman AD Kunz R What is “quality of evidence” and why is it important to clinicians? *BMJ* 2008; 336:995–998. 18456631 10.1136/bmj.39490.551019.BE PMC2364804

