# Supplementary material for: Benign Prostatic Hyperplasia and the Risk of Prostate Cancer and Bladder Cancer: A Meta-Analysis of Observational Studies
Source: Medicine (Baltimore). 2016 May 6;95(18):e3493. doi: 10.1097/MD.0000000000003493 (PMC4863764; doi:10.1097/MD.0000000000003493)
Supplement: Supplemental Digital Content [file medi-95-e3493-s001.pdf]

Supplemental Digital Content-Table 1. Quality assessment of included case-control studies

	Selection				Comparability	Exposure		
	Is the case definition adequate?	Representativeness of the cases	Selection of Controls	Definition of Controls	Comparability of cases and controls on the basis of the design or analysis	Ascertainment of exposure	Same method of ascertainment for cases and controls	Non-Response rate
Armenian 1974	1	1	0	1	2	1	1	1
Mishina 1985	1	1	0	1	2	1	1	0
Simons 1993	1	1	0	1	2	1	1	0
Wei 1994	1	0	0	1	1	1	1	0
Wang 1996	1	0	1	1	1	1	1	0
Zhu 1996	1	0	0	1	2	1	1	1
An 2000	1	0	1	1	2	1	1	0
Coker 2004	1	1	1	1	2	1	1	0
Liu 2007	1	0	0	1	2	1	1	0
Albanes 2011	1	1	0	1	2	1	1	1
Buckley 2011	1	1	0	1	1	0	1	1
Chornokur 2012	1	0	0	1	1	0	0	1
Honda 1988	1	1	1	1	2	1	1	0
Hung 2013	1	1	1	1	1	1	1	1
Monnsen 1982	1	0	0	1	1	1	1	0
Nakata 1995	1	1	1	1	1	0	1	1

Supplemental Digital Content-Table 2. Quality assessment of included cohort studies

	Selection				Comparability	Outcome		
Study	Representativeness of the exposed cohort	Selection of the non exposed cohort	Ascertainment of exposure	Demonstration that outcome of interest was not present at start of study	Comparability of cohorts on the basis of the design or analysis	Assessment of outcome	Was follow-up long enough for outcomes to occur	Adequacy of follow up of cohorts
Greenwald 1974	1	1	1	1	1	1	1	1
Hartman 1998	1	0	1	0	1	0	1	1
Armenian 1974	0	1	1	1	1	1	1	1
Chokkalingam 2003	1	0	1	1	1	1	1	1
Orsted 2011	1	1	1	1	2	1	1	1
Schenk 2011	1	1	1	1	2	1	1	1
Kang 2007	0	1	1	1	2	1	1	1
Zhou 2015	1	0	0	1	1	0	1	1
Tseng 2013	1	1	1	0	1	1	1	1

Supplemental Digital Content-Table 3. Sensitivity analysis of benign prostatic hyperplasia and the risk of urogenital cancers.

	Number of studies	Relative Risk (95%CI)	Heterogeneity
**Prostate cancer**			
Excluding studies with high risk of bias*	17	2.84 (1.75, 4.60)	I^2 = 97%; *P* < 0.00001
Excluding studies without matching or adjusting any factor	18	3.16 (1.94, 5.15)	I^2 = 97%;*P* < 0.00001
Excluding single-arm cohort studies	19	3.07 (1.94, 4.85)	I^2 = 97%; *P* < 0.00001
Excluding studies which did not report the detailed definition of BPH	9	2.22 (0.98, 5.05)	I^2 = 89%; *P* < 0.00001
**Bladder cancer**			
Excluding studies with high risk of bias*	4	1.70 (1.31, 2.22)	I^2 = 52%; *P* = 0.10
Excluding studies without matching or adjusting any factor	4	1.84 (1.62, 2.09)	I^2 = 0%; *P* = 0.54
Excluding studies undertaken in patients with diabetes	5	1.71 (1.22, 2.40)	I^2 = 38%; *P* = 0.17
Excluding single-arm cohort studies	9	3.07 (1.94, 4.85)	I^2 = 97%; *P* < 0.00001
Excluding studies which did not report the detailed definition of BPH	2	2.08 (1.47, 2.95)	I^2 = 75%; *P* = 0.0.4

*High risk of bias was defined as having a Newcastle-Ottawa Scale score < 7 points.

CI: Confidence Interval

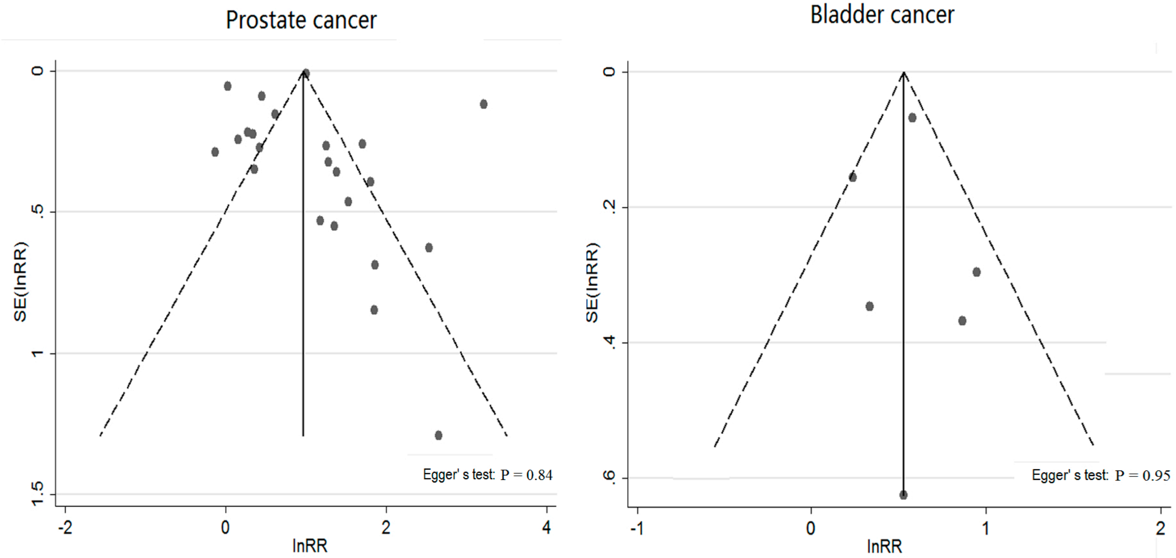

Figure. Evaluation of the risk of publication bias

Reference of included studies

S1. Hartman T, Albanes D, Pietinen P, et al. The association between baseline vitamin E, selenium, and prostate cancer in the Alpha-Tocopherol, Beta-Carotene Cancer Prevention Study. Cancer Epidemiology Biomarkers & Prevention. 1998; 7: 335-340

S2. Orsted D, Bojesen S, Nielsen S, et al. Association of clinical benign prostate hyperplasia with prostate cancer incidence and mortality revisited: A nationwide cohort study of 3 009 258 men. European Urology. 2011; 60: 691-698

S3. Schenk JM, Kristal AR, Arnold KB, et al. Association of symptomatic benign prostatic hyperplasia and prostate cancer: results from the prostate cancer prevention trial. American Journal of Epidemiology. 2011; 173: 1419 -1428

S4. Kang D, Chokkalingam A, Gridley G et al. Benign prostatic hyperplasia and subsequent risk of bladder cancer. British Journal of Cancer. 2007; 96: 1475-1479

S5. Tseng C. Benign prostatic hyperplasia is a significant risk factor for bladder cancer in diabetic patients: A population-based cohort study using the National Health Insurance in Taiwan. BMC Cancer. 2013; 13: 7

S6. Greenwald P, Kirmss V, Polan A, et al. Cancer of the prostate among men with benign prostatic hyperplasia. Journal of the National Cancer Institute. 1974; 53: 335-340

S7. Wei Q, Tang X, Yang Y, et al. A case-control study of the risk factors for prostate cancer. Journal of West China University of Medical Sciences 1994; 25: 87-90

S8. An N, Pang W, Liu Y. Case-control Study on the Relationship Between Prostate Cancer and History of Prostatic Diseases. Chin J Prev Contr Chron Non-commun Dis 2000; 8: 214-215

S9. Albanes D, Weinstein SJ, Snyder K, et al. Circulating 25-hydroxyvitamin D and prostate cancer risk in a large male cohort. Cancer Research. 2010; 70: S2796

S10. Coker A, Sanderson M, Zheng W, et al. Diabetes mellitus and prostate cancer risk among older men: Population-based case-control study. British Journal of Cancer. 2004; 90: 2171-2175

S11. Mommsen S, Aagaard J, Sell A. An epidemiological case-control study of bladder cancer in males from a predominantly rural district. Eur J Cancer Clin Oncol. 1982; 18: 1205-1210

S12. Mishina T, Watanabe H, Araki H, et al. Epidemiological study of prostatic cancer by matched-pair analysis. Prostate. 1985; 6: 423-436

S13. Zhou J, Kelsey KT, Smith S, et al. Lower Urinary Tract Symptoms and Risk of Bladder Cancer in Men: Results From the Health Professionals Follow-up Study. Urology. 2015;85:1312-8

S14. Chokkalingam A, Nyren O, Johansson J et al. Prostate carcinoma risk subsequent to diagnosis of benign prostatic hyperplasia: a population-based cohort study in Sweden. Cancer. 2003; 98: 1727-1734

S15. Liu C, Yang Z, Li S. Prostate Diseases, Sexuality and Prostate Cancer: a Case-control Study. Medical Journal of Wuhan University. 2007; 28: 219-221

S16. Armenian HK, Lilienfeld AM, Diamond EL, et al. Relation between benign prostatic hyperplasia and cancer of the prostate. A prospective and retrospective study. Lancet. 1974; 2: 115-117

S17. Simons BD, Morrison AS, Young RH, et al. The relation of surgery for prostatic hypertrophy to carcinoma of the prostate. American Journal of Epidemiology. 1993; 138: 294-300

S18. Chornokur G, Han G, Tanner R et al. Risk factors of prostate cancer in African American men. Cancer Research. 2012; 72: S3592

S19. Buckley B, Lapitan M, Simpson C, et al. Risk of prostate cancer associated with benign prostate disease: a primary care case-control study. British Journal of General Practice. 2011; 61: 684-691

S20. Wang R, Gu L, Ann H, et al. Sexual behavior, non-cancerours diseases and prostatic cancer : a case-control study. Chin J Urol. 1996; 17: 481-487

S21. Hung S, Lai S, Tsai P, et al.: Synergistic interaction of benign prostatic hyperplasia and prostatitis on prostate cancer risk. British Journal of Cancer. 2013; 108: 1778-1783

S22. Zhu K, Stanford JL, Daling JR, et al. Vasectomy and prostate cancer: a case-control study in a health maintenance organization. Am J Epidemiol. 1996; 144: 717-722

S23. Honda G, Bernstein L, Ross R, et al. Vasectomy, cigarette smoking, and age at first sexual intercourse as risk factors for prostate cancer in middle-aged men. British Journal of Cancer. 1988; 57: 326-331

S24. Nakata S, Sato J, Ohtake N, et al. Epidemiological study of risk factors for bladder cancer. Hinyokika Kiyo.1995; 41: 969-977
